# ‘Guard’ Workout: Can a Lifeguard-Specific High-Intensity Functional Training Reflect Rescue Demands?

**DOI:** 10.3390/jfmk11020218

**Published:** 2026-05-29

**Authors:** Isaac Ignacio-Rodríguez, Silvia Aranda-García, Marcos Sanmartín-Montes, Oscar Morales-Rejas, Martín Otero-Agra, Myriam Santos-Folgar, Fernando Zarzosa-Alonso, Roberto Barcala-Furelos

**Affiliations:** 1 REMOSS Research Group, Faculty of Education and Sport Sciences, Universidade de Vigo, 36005 Pontevedra, Spain; isaacrodriguezignacio@gmail.com (I.I.-R.); marcos.sanmartin@uvigo.es (M.S.-M.); martinoteroagra@gmail.com (M.O.-A.); m.santos.folgar@gmail.com (M.S.-F.); fzarzosa@uvigo.es (F.Z.-A.); 2 GRAFIS Research Group, Institut Nacional d’Educació Física de Catalunya (INEFC), University of Barcelona, 08038 Barcelona, Spain; saranda@gencat.cat; 3 School of Nursing of Pontevedra, Universidade de Vigo, 36005 Pontevedra, Spain; 4 Cidega, Institute for Research in Sport Sciences, Universidade de Vigo, 36005 Pontevedra, Spain

**Keywords:** lifeguard, fitness, water rescue, HIFT, drowning prevention, cardiopulmonary resuscitation

## Abstract

**Objectives**: In lifeguarding, when prevention fails, rapid and safe rescue is essential to prevent submersion and enable appropriate care. These operations demand high physical fitness. High-Intensity Functional Training (HIFT), such as CrossFit^®^, has become a widely used training model. This study examined the integration of HIFT into lifeguard training to enhance preparedness for aquatic rescues. The aim was to analyse the external and internal load generated by a lifeguard-specific HIFT protocol and to assess its utility both as a training method and as a fitness evaluation tool. **Methods**: Twenty-seven lifeguards completed a 15 min HIFT protocol in an AMRAP (As Many Rounds As Possible) format with four stations: running, swimming with a tow, victim extraction, and cardiopulmonary resuscitation (CPR). Variables measured included time per round (TRound), number of completed rounds, maximum and average heart rate (MaxHR, AvgHR), post-exercise blood lactate, rating of perceived exertion (RPE) per round and overall, and CPR quality (Q-CPR). **Results**: TRound ranged from 238 to 268 s, with significant differences between consecutive rounds, though remaining at submaximal levels. Most participants (74%) completed three rounds, and none completed four. AvgHR was 164 bpm (82% of MaxHR). RPE increased significantly across rounds (*p* < 0.001), reaching 9 post-AMRAP (*p* < 0.001). Post-AMRAP blood lactate was 15.50 mmol/L (*p* < 0.001), indicating high physiological stress. Despite accumulated fatigue, Q-CPR remained consistently high, ranging from 86% to 94%, with no significant differences across rounds. **Conclusions**: The results of this pilot study suggest that physically active lifeguards can sustain a controlled, high-intensity effort at submaximal heart rates and high perceived exertion without compromising CPR quality. These preliminary findings support the feasibility and potential utility of a lifeguard-specific HIFT programme structured as an AMRAP for high-intensity training and ecological fitness assessment in rescue-specific conditions; formal validation studies are needed before broader recommendations can be made.

## 1. Introduction

Lifeguards are key players in the fight against drowning. Their primary role is prevention [1]. When this fails, the next links in the chain of survival require a rapid and safe rescue to prevent submersion, remove the person from the water, and provide the necessary care [2].

Rescue is the most physically demanding task for lifeguards, during which they sustain efforts characterised by heart rate responses typically exceeding the anaerobic threshold yet remaining below maximum capacity—a pattern consistent with submaximal supra-threshold exertion [3,4]. Therefore, physical preparation plays a crucial role in the success of the rescue [5], potentially making the difference between life and death, while also influencing the lifeguard’s own safety given the hostile and uncertain conditions in which they operate [6,7].

Due to the nature of their work, lifeguards alternate periods of relative inactivity with sudden bursts of high-intensity effort, which requires a high level of fitness to meet the demands of aquatic rescue [5]. For this reason, training focused on high-intensity cardiovascular and strength work, specifically tailored to aquatic rescue and presented in a competitive and motivating format, may contribute to their preparation both during training stages and working periods, when time to stay fit is limited. The AMRAP format, characteristic of HIFT-based programmes such as CrossFit^®^, offers structural features that may be well suited to the intermittent and cumulative demands of lifeguard training, though direct evidence in aquatic rescue populations remains limited [8,9,10,11].

We hypothesised that a lifeguard-specific HIFT protocol structured as an AMRAP would induce high physiological stress—as reflected by heart rate, blood lactate, and rating of perceived exertion—while preserving CPR quality across successive rescue rounds, consistent with the demands of real professional rescue scenarios.

The aim of this study is to analyse the external and internal load of a HIFT programme designed for professional lifeguards, exploring its applicability both as a training stimulus and as a method for assessing physical fitness under functional conditions. Importantly, while HIIT is characterised by repeated bouts of vigorous effort interspersed with rest or low-intensity recovery, HIFT incorporates functional, multi-joint movements performed at high intensity with constantly varied exercise selection—features that align with the task-specific, unpredictable nature of aquatic rescue [8,11].

## 2. Materials and Methods

### 2.1. Study Design

A descriptive pilot design was used to analyse the response, in terms of rescue time, CPR quality, perceived fatigue, and accumulated lactate, in professional aquatic lifeguards during a 15 min training circuit using the AMRAP training structure. The pilot design was selected because no prior data existed on the physiological responses to this novel protocol in lifeguards, precluding a formal a priori power calculation; the primary aim was to establish feasibility and characterise the physiological load under real environmental conditions, generating effect size estimates to inform future confirmatory studies.

### 2.2. Participants

A convenience sample of 27 lifeguards (5 women and 22 men) was selected, all of whom voluntarily participated in this pilot study. As inclusion criteria, participants had to be actively working as lifeguards or enrolled in a training programme with at least two months of specific training. Regarding CPR, all participants were required to have received specific training within the previous two months in accordance with the European Resuscitation Council Guidelines 2025 [12,13]. Specific experience with CrossFit^®^ or HIFT-based methodologies was not an inclusion criterion and was not systematically collected; this represents a limitation, as prior HIFT experience may have influenced the pacing strategies observed.

Additionally, participants were required to be physically active, meeting or exceeding the World Health Organization (WHO) recommendation of 150 min of moderate physical activity per week [14]. This threshold was selected as a minimum baseline criterion to ensure participants were not sedentary, rather than to characterise professional fitness levels; objective indicators such as VO_2_max estimates or rescue-specific benchmarks were not recorded, which is acknowledged as a limitation. The sample was predominantly male (22 men vs. 5 women), which may limit the generalisability of findings given evidence of sex-related differences in physiological responses to high-intensity exercise. The study was approved by the Ethics Committee of the Faculty of Education and Sport Sciences of the University of Vigo (code 07-151025) and was conducted in accordance with the Declaration of Helsinki. All participants provided written informed consent prior to participation.

### 2.3. High-Intensity Functional Training: ‘Guard’ Workout

**Figure 1:**
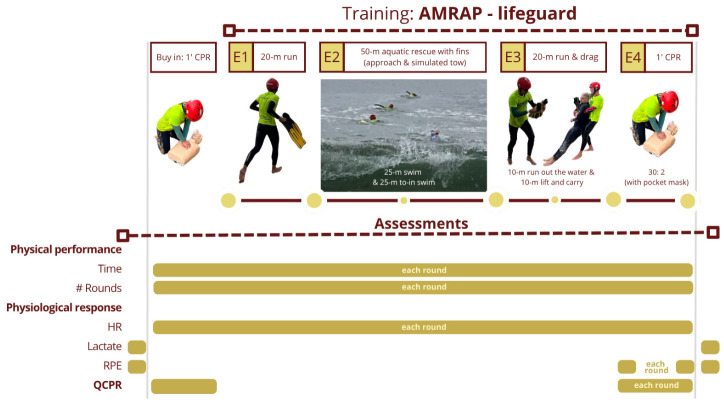
Flowchart of the ‘Guard’ Workout study design and assessments.

An AMRAP training session was carried out following the CrossFit^®^ methodology. During a 15 min work period, lifeguards began with 1 min of CPR (referred to as a “buy-in” in CrossFit^®^ terminology). During the remaining time, they performed as many rounds as possible of the following training circuit with four specific exercises (E) (Figure 1): E1—20 m run: a 20 m run across the sand to the water. E2—50 m aquatic rescue with fins: a 25 m approach swim with fins followed by a 25 m simulated tow of an unconscious victim (without an actual victim) back to shallow water, with fin removal prior to exiting the water. E3—20 m run and drag on sand: a 10 m run out of the water followed by a 10 m uphill victim drag on the sand, using a simulated victim represented by a peer of similar body mass to the participant. E4—1 min CPR: one minute of CPR using cycles of 30 compressions and 2 ventilations with a pocket mask on a manikin.

Before the session, lifeguards completed a standardised 10 min warm-up consisting of 5 min of progressive jogging and dynamic mobility exercises (hip circles, arm swings, and leg swings), followed by 5 min of sport-specific preparation, including shallow-water entries, short low-intensity swim sets, and two practice CPR cycles on the manikin. Movement execution speed and transition times between stations were verbally controlled by trained research staff. CPR was performed in cycles of 30 compressions and 2 ventilations with a pocket mask, targeting a compression rate of 100–120/min and depth of 5–6 cm per ERC 2025 guidelines [12]. The 15 min AMRAP duration was selected based on HIFT literature using comparable formats in emergency professional populations [15] and the estimated time window of a prolonged or multi-casualty beach rescue scenario.

### 2.4. Variables and Measurement Procedures

Three groups of variables were assessed (Figure 1):

1. Physical performance: number of circuit rounds and time per round (TRound) in seconds (s).
2. Physiological response:
  (a) Maximum heart rate (MaxHR) and average heart rate (AvgHR), monitored using the Polar Verity Sense heart rate monitor and the Polar Team App (Polar Electro OY, Kempele, Finland) [16].
  (b) Capillary blood lactate (mmol/L) was assessed using Lactate Scout (SensLab GmbH, Leipzig, Germany) [17], with an accuracy of ±3%. It was measured before and 3 min after completing the training [18], consistent with established post-exercise peak lactate windows following intense intermittent exercise [19]. The first drop of blood was discarded in both measurements.
  (c) Rating of perceived exertion (RPE), using the Borg CR-10 scale (all participants received a brief standardised explanation of the scale, including verbal anchors [0 = rest; 10 = maximal exertion], and practised one familiarisation rating before the warm-up began) [20]. It was measured at the beginning (before buy-in 1′CPR), after each round (after E4: 1′CPR) and at the end of the training session.
3. CPR quality (Q-CPR) as a percentage (%): assessed during the “buy-in” [baseline], and in each training round, monitored using a Laerdal Resusci Anne^®^ manikin (Laerdal Medical, Stavanger, Norway) [21] connected to the Laerdal Resusci Anne^®^ QCPR^®^ Trainer app for iOS (Laerdal Medical, Stavanger, Norway) [22]. This application provides a standardised score for CPR quality based on the ERC Guidelines 2025 [12,13].
4. Demographic variables: age (years), weight (kg), height (m), and sex were recorded to describe the participant sample.

### 2.5. Location, Environmental Conditions, and Lifeguard Equipment (Figure 2)

The study was conducted at Mogor Beach, in Marín (Pontevedra, Galicia, Spain), with coordinates 42.3856° N and −8.7202°. The weather conditions were as follows: light showers, air temperature between 10 °C and 15 °C, moderate northwest winds (approximately 15–20 km/h), and a moderate swell, with wave heights ranging from 1.4 to 1.8 m. The test was carried out at high tide, which created an intense breaking zone that made both water entry and exit more difficult.

For safety reasons, all participants wore wetsuits (3–4 mm), protective helmets, and swimming fins. In addition, three members of the research team trained in lifeguarding and two emergency nurses supervised the entire protocol to ensure participants’ safety.

**Figure 2:**
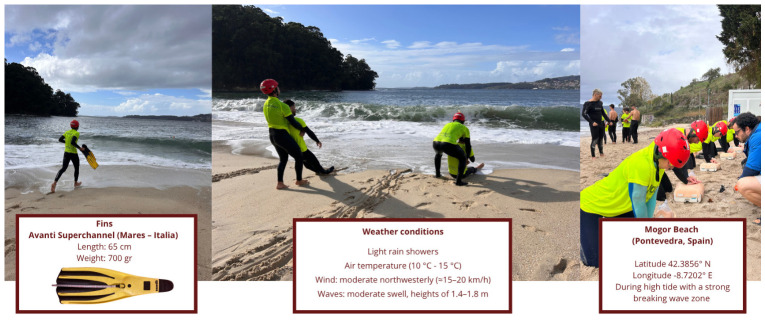
Location, environmental conditions, and lifeguard equipment used during the ‘Guard’ Workout.

### 2.6. Statistical Analysis

All analyses were performed with IBM SPSS Statistics software version 21 for Windows (Armonk, NY, USA). For the description of variables, continuous variables were described through measures of central tendency (median) and dispersion (interquartile range or IQR). Categorical variables were described through absolute and relative frequencies. Normality was assessed using the Shapiro–Wilk test. For intragroup comparisons, the Wilcoxon signed-rank test was used for two-point comparisons and the Friedman test with Bonferroni post hoc correction for comparisons across more than two time points. Effect sizes are reported as r (=|Z|/√N) for Wilcoxon comparisons and Kendall’s W (=χ^2^/[N × (k − 1)]) for Friedman tests, interpreted as small (r ≥ 0.10; W ≥ 0.10), moderate (r ≥ 0.30; W ≥ 0.30), or large (r ≥ 0.50; W ≥ 0.50). In all analyses, a value of *p* = 0.05 was assigned.

## 3. Results

Participant characteristics were: median age of 20 years (IQR 20–21), median height of 176 cm (IQR 171–179), and median weight of 72 kg (IQR 63–77).

### 3.1. Physical Performance

The median TRound for round 1 was 238 s (IQR 222–255), for round 2 was 250 s (IQR 240–299), for round 3 was 268 s (IQR 239–343), and for round 4 was not computable since no participant completed this round (Table 1). Significant increases in TRound were observed between rounds 1 and 2 (*p* = 0.004) and between rounds 2 and 3 (*p* < 0.001), while no statistically significant difference was found between rounds 1 and 3 (*p* = 0.055). All 27 lifeguards (100%) completed rounds 1 and 2; 20 (74%) completed round 3. Twenty participants (74%) started round 4 but none finished it. A Friedman test across the three completed rounds revealed a significant overall effect (chi^2^(2) = 31.61, *p* < 0.001, Kendall’s W = 0.61, large effect). Pairwise Bonferroni-corrected comparisons showed significant increases in TRound between rounds 1 and 2 (*p* < 0.001, r = 0.74, large effect; *n* = 26), between rounds 2 and 3 (*p* < 0.001, r = 0.72, large effect), and between rounds 1 and 3 (*p* < 0.001, r = 0.85, large effect).

### 3.2. Physiological Response: Heart Rate, Blood Lactate, and RPE

The median peak MaxHR was 185 bpm (IQR 177–189), representing 93% (IQR 90–95) of the estimated maximum heart rate. The AvgHR was 164 bpm (IQR 158–173), corresponding to 82% of MaxHR (IQR 79–86) (Table 2).

Pre-AMRAP blood lactate (baseline) was 1.50 mmol/L (IQR 1.30–2.20). Post-AMRAP blood lactate was 15.50 mmol/L (IQR 14.20–16.75; *p* < 0.001) (Table 3). All participants began from absolute rest. The Wilcoxon comparison yielded a large effect size (r = 0.87, *n* = 27). RPE 0 and finished at near-maximal effort (RPE 9, IQR 8–9; *p* < 0.001). Across rounds, RPE increased linearly from 6 (IQR 5–7) at the end of round 1 to 9 (IQR 9–10) at the end of round 4 (Friedman chi^2^(4) = 74.55, *p* < 0.001, Kendall’s W = 0.93, large effect; *n* = 20; pre–post: r = 0.87, *p* < 0.001).

### 3.3. CPR Quality in Percentage (%)

Q-CPR at the buy-in (baseline) was 94% (IQR 86–99). In round 1: 87% (IQR 82–99); round 2: 86% (IQR 82–93); round 3: 87% (IQR 85–97); round 4: 94% (IQR 87–99). No statistically significant differences were observed across rounds (Friedman chi^2^(3) = 5.29, *p* = 0.152, Kendall’s W = 0.10, negligible effect; *p* > 0.05).

**Figure 3:**
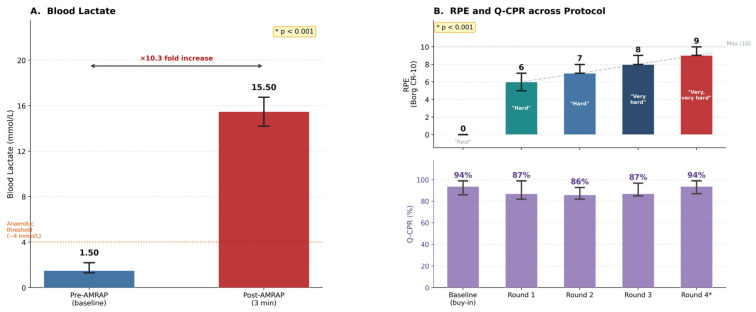
Physiological effort variables across the ‘Guard’ Workout protocol. (**A**) Blood lactate concentration pre- and post-AMRAP (median ± IQR). ((**B**), upper panel) RPE (Borg CR-10 scale) from baseline through each round (median ± IQR); dashed line represents the linear trend across rounds. ((**B**), lower panel) Q-CPR (%) at baseline and across rounds (median ± IQR). * *p* < 0.001.

Figure 3 integrates the three key physiological effort variables recorded throughout the protocol. As shown in Panel A, blood lactate increased more than tenfold from pre- to post-AMRAP (1.50 to 15.50 mmol/L; *p* < 0.001), confirming the high anaerobic metabolic demand generated by the ‘Guard’ Workout. Panel B illustrates the concurrent evolution of perceived exertion and CPR quality across the protocol stages. While RPE increased progressively and significantly from rest (0 at baseline) to near-maximal exertion (9 at Round 4; *p* < 0.001), Q-CPR remained consistently high throughout all stages, ranging from 86% to 94%, with no statistically significant differences across rounds (*p* > 0.05).

## 4. Discussion

The aim of this study was to evaluate a high-intensity training and fitness assessment programme for lifeguards that included all the typical elements of their interventions (running, approach swimming, towing simulation, extraction and CPR), aimed at resolving incidents in the aquatic environment. The main findings were:

The relatively modest increase in round time across completed rounds (from 238 s in Round 1 to 268 s in Round 3, approximately 13%), combined with submaximal heart rate responses and progressively increasing yet controlled RPE values, is consistent with a self-regulated pacing pattern. However, as pacing behaviour was not directly assessed through biomechanical or psychophysiological measures, this interpretation remains inferential.

These findings indicate that participants were able to sustain a substantial glycolytic metabolic contribution—as indexed by blood lactate (r = 0.87, large effect), which reflects the balance between production and clearance during intermittent exercise rather than serving as a sole marker of fatigue—alongside increasing perceived effort (r = 0.87, large effect), without any measurable deterioration in CPR quality (Kendall’s W = 0.10, negligible effect).

Faced with the challenge of the most demanding task for a rescuer—rescue [3]—the specific training of a lifeguard must be based on solid physical and technical training that allows him to carry out rescues quickly and effectively [23]. Several studies have shown that a water rescue involves a submaximal physical demand, with efforts above the anaerobic threshold in terms of heart rate [24] and high lactic demands [4,24], in addition to a notable importance of strength. Similar results were observed in this study, in addition to RPE values indicating submaximal efforts.

De Oliveira et al. [5] characterised the physical profile of military lifeguards in Brazil. The results showed significant differences in jumping, sprinting and agility tests, among other variables. These findings are useful for measuring performance and its evolution over time. However, our study is based on the need to complement the preparation of lifeguards with tests that include ecological components, capable of evaluating technical performance in an integrated way, together with the management of physical capacities in a changing and realistic environment. In fact, it is estimated that the swimming experience in a realistic and natural environment can explain up to 18% of the performance [25].

This raises the question of whether it is possible to implement training in a realistic environment that incorporates the essential components of water rescue while simultaneously serving as a standardised performance measurement tool—an objective that the present study sought to address.

In recent years, modalities based on High-Intensity Functional Training (HIFT) have gained substantial global traction as an evidence-informed training model [9,26], with documented experiences integrating them with the specific occupational tasks of professional groups related to emergencies, such as police [15] or firefighters [10].

In particular, CrossFit^®^ or Hyrox^®^ based approaches are associated with various physiological adaptations—metabolic, cardiovascular, and muscle strength— [27,28,29,30] and often require maintaining a high intensity, especially when seeking to perform as many repetitions as possible in a fixed time, a format known as “As Many Rounds As Possible”. This can align with simulating a multi-casualty incident, where more than one victim needs to be rescued, which in lifeguarding is known as a “worst-case scenario.”

A key aspect for lifeguards is self-knowledge and self-regulation of effort since, due to the casuistry of their working day, they may find themselves facing several rescues on the same day. In this sense, total round time increased by only 30 s from the first to the last round, with an approximate average of 4 min per round for a total distance of 100 m (between sea and land). Similar results were observed in a study with surf instructors, in which similar times were maintained for each rescue over a comparable distance, although surfers did not exhibit significant variations between rounds [31]. This possibly occurred due to the knowledge of the motor commitment time (15′), which allowed them to self-regulate, performing the AMRAP incrementally, but without reaching 100% of their maximum capacity, as reflected in the RPE, which increased as the number of repetitions progressed until reaching a submaximal intensity (9 of 10). This would prevent collapse during activity, which could put them at risk in a real rescue situation in the water. This form of regulation during the different phases and repetitions of the test is a concept that, in the field of sport, is known as “pacing strategies” [32] and is widely used in competitive swimming [33]. But why is it relevant for rescuers to conserve some of their energy at the end of the rescue? In addition to the aforementioned casuistry, we must bear in mind that the next link in the chain of survival of drowning is the provision of care to the rescued person [2]. Among them, CPR is the most critical technique that should be applied since it is one of the factors that will determine the chances of survival of the drowning person [34]. Previous studies have shown how pre-rescue fatigue can reduce the quality of CPR [35,36]; therefore, attenuating this decrease is a relevant objective not only for training, but also for the chances of survival in out-of-hospital cardiac arrest. This study shows that physically active lifeguards can maintain a good quality of CPR despite the accumulated fatigue.

### Practical Applications and Limitations

This study provides preliminary evidence on the feasibility of a high-intensity training programme for lifeguards, inspired by the CrossFit^®^ methodology, which can be implemented in real professional contexts, either as part of maintaining fitness during the work season, in which lifeguards usually have a reduced time to train, or as part of the tune-up before the work season. Along these lines, the figure of the “tactical athlete” has emerged in recent years, defined as those professionals who require unique physical training strategies to optimise their occupational physical performance [37]. Specifically, when it comes to CPR quality, scientific evidence has shown relationships between strength [38] and anaerobic endurance [39] and CPR quality.

In addition, this training model can be offered in a competitive format, providing ecological validity to sports rescue competitions in which real techniques are applied and adjusted to the professional demands of rescue and resuscitation, including CPR.

Despite the promising findings, this study has certain limitations. First, the sample size was relatively small and not representative of all first responders, which could limit the generalizability of the results. In addition, the study design was pilot and descriptive, implying that no comparisons were made with control groups or different training methods. No test–retest reliability assessment was conducted, and potential order or learning effects across rounds cannot be excluded. Heart rate data were unavailable for seven participants due to technical difficulties with the optical sensor during aquatic segments. Additionally, the aquatic tow was performed without an actual victim to ensure participant safety, which likely underestimates the physiological load of a real rescue. Specific environmental conditions on the day of the test, such as temperature and sea state, could also have influenced the participants’ performance, suggesting the need to replicate the study in different contexts and conditions. Furthermore, while the real-sea setting enhances ecological validity, wave variability, tidal conditions, and wind may have differentially affected individual rescue times and physiological responses in ways that are difficult to fully control or replicate. Finally, although physiological and performance variables were measured, other psychological or motivational factors that could influence the performance of the rescuers during training and actual rescue situations were not evaluated. Future studies should explore whether HIFT-based rescue protocols produce different physiological or performance responses across fitness levels, training backgrounds, and sexes, given the highly imbalanced sex ratio of the present sample (22 men vs. 5 women).

## 5. Conclusions

The results of this pilot study suggest that physically active lifeguards can sustain a controlled, high-intensity effort at supra-anaerobic-threshold intensity with high perceived exertion across multiple rescue rounds without compromising CPR quality. These preliminary findings support the feasibility and potential utility of a lifeguard-specific HIFT protocol structured as an AMRAP, both as a high-intensity training model applicable to real professional contexts and as a candidate tool for ecological fitness assessment under rescue-specific conditions. However, formal validation through reliability and criterion validity studies is required before broader implementation can be recommended.

## Figures and Tables

**Table 1:** Number of lifeguards who finished the rounds and time per round.

Round Nº	LGS	LGF	TRound (s)	TRounds vs. Previous Rounds
	*n* (%)	*n* (%)	Median (IQR)	
1	27 (100%)	27 (100%)	238 (222–255)	
2	27 (100%)	27 (100%)	250 (240–299)	R2 vs. R1 *p* = 0.004
3	27 (100%)	20 (74%)	268 (239–343)	R3 vs. R2 *p* < 0.001 R3 vs. R1 *p* = 0.055
4	20 (74%)	0 (0%)	- *	

Round Nº, round number. s, seconds. LGS, lifeguards started. LGF, lifeguard finished. *n*, number of lifeguards. TRound, time per round. * No times were recorded because none of the lifeguards completed Round 4.

**Table 2:** HR variables during AMRAP (*n* = 20; missing = 7).

	Median (IQR)
MaxHR (bpm)	185 (177–189)
MaxHR (%)	93 (90–95)
AvgHR (bpm)	164 (158–173)
AvgHR (%)	82 (79–86)

HR: heart rate; MaxHR: maximum heart rate; AvgHR: average heart rate; bpm: beats per minute.

**Table 3:** Physiological effort variables.

	Median (IQR)
Lactate	
Pre-AMRAP blood lactate (baseline) (mmol/L)	1.50 (1.30–2.20)
3′Post-AMRAP blood lactate (mmol/L)	15.50 (14.20–16.75)
	*p* < 0.001 (r = 0.87)
RPE	
Pre-AMRAP RPE (baseline)	0 (0–0)
Post-AMRAP RPE	9 (8–9)
	*p* < 0.001 (r = 0.87)
After Round 1 RPE	6 (5–7)
After Round 2 RPE	7 (7–8)
After Round 3 RPE	8 (8–9)
After Round 4 RPE *	9 (9–10)
	*p* < 0.001 (W = 0.93)

mmol/L: millimoles per litre; RPE: Rating of Perceived Exertion (0–10 scale). * No lifeguard completed Round 4; RPE was measured at the last exercise reached.

## Data Availability

The data presented in this study are available on request from the corresponding author. The data are not publicly available due to containing information protected by the Spanish Organic Law on Personal Data Protection and Digital Rights Guarantee.

## Abbreviations

The following abbreviations are used in this manuscript:

AMRAP	As Many Rounds As Possible
AvgHR	Average Heart Rate
bpm	Beats Per Minute
CPR	Cardiopulmonary Resuscitation
E	Exercise (Station)
ERC	European Resuscitation Council
HIFT	High-Intensity Functional Training
IQR	Interquartile range
LGF	Lifeguards finished
LGS	Lifeguards started
MaxHR	Maximum heart rate
Q-CPR	CPR Quality Score (%)
RPE	Rating of Perceived Exertion
TRound	Time Per Round (Seconds)
WHO	World Health Organization
